# Workplace violence against doctors in China: A case analysis of the Civil Aviation General Hospital incident

**DOI:** 10.3389/fpubh.2022.978322

**Published:** 2022-08-30

**Authors:** Yu Xiao, Na Du, Jia Chen, Ya-lan Li, Qin-ming Qiu, Shao-yi Zhu

**Affiliations:** ^1^Psychosomatic Medical Center, The Fourth People's Hospital of Chengdu, Chengdu, China; ^2^Psychosomatic Medical Center, The Clinical Hospital of Chengdu Brain Science Institute, MOE Key Lab for Neuroinformation, University of Electronic Science and Technology of China, Chengdu, China; ^3^Department of Psychiatry, Huzhou Third People's Hospital, Huzhou, China; ^4^Department of Psychiatry, Shantou University Mental Health Center, Shantou, China

**Keywords:** doctor, hospital violence, occupational safety, social media, workplace violence

## Abstract

**Background:**

Violence against health professionals is a global public health problem. In 2019, a doctor was killed in Civil Aviation General Hospital (CAGH), which triggered national discussion about hospital violence. Sina Weibo, the Chinese version of Twitter, played an important role in this public discussion. The CAGH incident provides us with an opportunity to explore how social media was used in the discussion on violence against doctors.

**Methods:**

Using the built-in search engine of Sina Weibo, a data set containing 542 Chinese micro-blogs was established. Three keywords: Civil Aviation General Hospital, doctor, and knife were used to search for related posts between December 24th, 2019 and January 19th, 2020. We made a content analysis of the posts to investigate: Weibo users' demographics, views about the incident of CAGH, and measures to prevent hospital violence.

**Results:**

Overall, 89.3% of the posts were sent by individual Weibo users, and 10.7% by organizations. Among the individual users, doctors accounted for 27.4%, but only 1.0% came from the legal profession. In addition, 86.7% of the micro-blogs expressed sympathy for the attacked doctor, and 23.1% of the micro-blogs thought that the imperfect medical system was the main cause of the accident. Nearly half of the posts described their disappointment with the government and the society, and 58.6% of medical staff users expressed regret for engaging in medical work. Only 14.2% of micro-blogs put forward some constructive strategies to prevent hospital violence.

**Conclusion:**

Weibo users played an important role in spreading and discussing the CAGH incident. However, constructive measures to protect doctors were rarely mentioned, and legal opinions were not reflected in time. Hospital violence has caused public dissatisfaction with the government and weakened the professional confidence of medical staff. Occupational health and public health stakeholders must take effective measures to solve workplace violence against doctors.

## Introduction

Workplace violence (WPV) is considered as a worrying occupational health hazard (1). WPV refers to an individual's or group's socially unacceptable, aggressive (and sometimes destructive) behavior (2). In all cases of WPV, attacks on health professionals (HPs) account for almost a quarter (3). Specifically, WPV can be psychological or physical, including but not limited to verbal abuse, mobbing, stabbing or shooting (3). Serious physical violence may lead to injury or even death of the victim. The perpetrators of hospital violence are some patients, some patients' families, and some neither (4).Violence often happens after medical malpractice or even when the treatment results fail to meet patients' expectations (5). The data show that in the past 10 years, the incidence of WPV against HPs in China has been increasing, and it has become a serious and persistent social problem (6).

On December 24th, 2019, a patient's family named Sun cut off a doctor's head with a knife in the emergency room due to dissatisfaction with the treatment effect (7). The victim is Dr. Wen Yang, a female doctor at Civil Aviation General Hospital (CAGH) in Beijing. This case quickly triggered the public's condemnation of the perpetrator and the discussion of hospital violence. On January 16, 2020, Sun was sentenced to death (8). This incident is a shocking one, but it is not an isolated one. A meta-analysis in 2019 showed that among global HPs, 61.9% reported exposure to any form of WPV, 42.5% reported exposure to non-physical violence, and 24.4% reported experiencing physical violence in the past year (9). According to Chirico et al.'s (4) data, during the COVID-19 epidemic, HPs were exposed to different types of WPV, with prevalence rates ranging between 5.8%−44.4% for physical violence and 9.6%−97.6% for verbal violence. Recent research shows that the global prevalence of WPV by patients and visitors against HPs is high, especially in Asian countries, emergency department settings, and among doctors and nurse (9). A recent cross-sectional study (10) of 134 hospitals from 16 provinces in China reported that the prevalence of medical WPV was 65.8%, including physical violence (11.8%) and verbal violence (64.9%). The damage caused by WPV not only translates into physical and mental harm to HPs, but also translates into short-term and long-term high cost for the organization where violence occurs, which reduces the quality of care provided to all patients (11, 12). Serious physical violence against HPs, though not as common as verbal abuse, may attract more attention from the public and mass media. It shows the worst doctor-patient relationship, and also reveals some defects in legal and medical systems.

Cases like the CAGH incident usually happen suddenly, and it's difficult to study this topic through conventional methods, such as interviews. Therefore, the analysis of social media content can provide a new perspective for the study of WPV. Sina Weibo, or micro-blogs, with more than 500 million users by 2021, provides a variety of interactive communication mechanisms for the Chinese public to share information and exchange opinions (13). Existing research shows that when it comes to health-related topics, social media can promote mutual understanding and social progress (14). However, in the context of hospital violence in China, there are few studies on the role and nature of social media, such as who is actively spreading information and participating in discussions, and what is presented on Weibo. The CAGH incident provides us with an opportunity to discuss these issues and expand our knowledge of WPV. In this paper, by analyzing the posts about the CAGH incident shared by users on Sina Weibo, we can better understand the attitudes and needs of the public and organizations, and clarify the existing challenges faced by government departments and society in dealing with hospital violence.

## Methods

### Database and search strategy

The data of this study came from Sina Weibo, the largest social media platform in China; it enables users to send and receive short posts with limited characters, and retrieve text content by searching for specified keywords within a defined date range. Using this function, we collected the original Weibo posts related to the CAGH incident from December 24, 2019 to January 19, 2020. Micro-blogs re-posted by other users were excluded because they did not provide new information. December 24, 2019 was set as the starting date of our research, because the accident happened on that day. The perpetrator was sentenced to death on January 16, 2020. After January 19, 2020, no original posts related to the CAGH incident were retrieved, so this day was chosen as the end date of this study.

**Figure 1:**
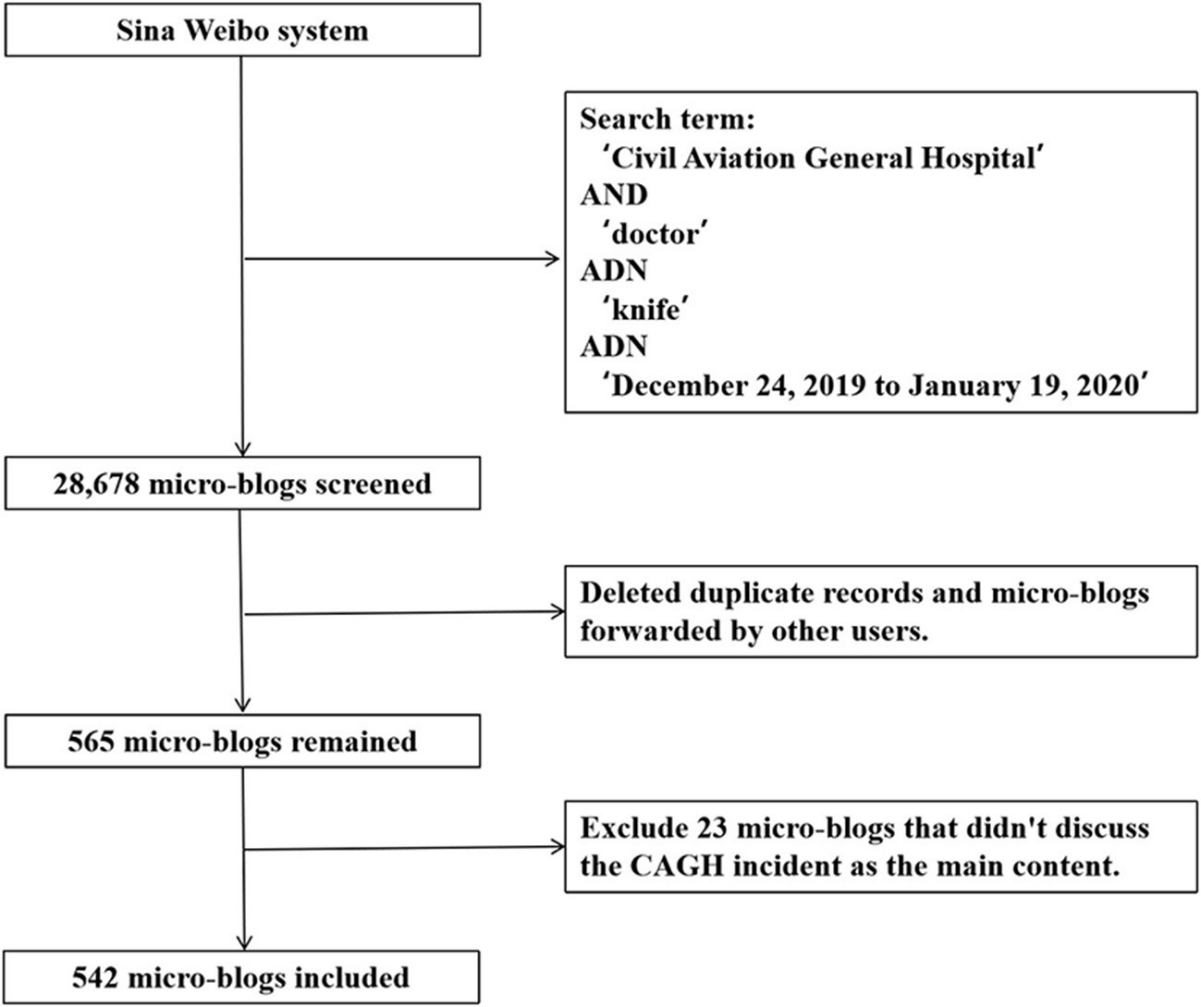
Flow chart of Weibo post extraction.

In order to make full use of micro-blogs related to the CAGH incident, we first conducted an exploratory search on Weibo to determine a list of related search terms. As the result of exploratory search, we identified three Chinese search terms: Civil Aviation General Hospital (“民航总医院”), doctor (“医生”), and knife (“刀”). The Octopus web crawler tool was used to search for predefined keywords. This tool has been proved to be effective in identifying the most relevant micro-blogs containing set keywords. In addition to the collected text content, we also sourced the metadata of each Weibo post, including user attributes and publishing time. Accounts of media and government were verified by Weibo to be “official” at registration by submitting relevant documents of their organizations for verification. The preliminary retrieval results produced a data corpus consisting of 28,678 Chinese micro-blogs. In order to delete duplicate records and micro-blogs forwarded by other users, we used the advanced search function built in Weibo, and finally retained 565 micro-blogs. Then, our researchers manually screened all micro-blogs to exclude 23 posts that did not discuss the CAGH incident as the main content. By enlisting these criteria, 542 micro-blogs constituted the data set of our study. Figure 1 features the flow chart that details the document extracting process.

### Variable coding and data analysis

To prepare for quantitative analysis, the extracted files were translated and encoded into numbers and string variables in standard data format. A tentative coding scheme was first developed based on preliminary analysis of a random training subset of 100 posts using open coding by one author (YX) and iteratively refined through independent analysis of another 100 training posts by authors YX, ND, and QQ, and discussion from the team. According to the analysis results, we built a coding framework. This framework included the following 12 questions: (1) Is the sender an individual or organizational user? (2) If it is posted by an organization, what type of organization is it? (3) What is the background of the individual user? (4) Whether to show sympathy for the dead doctor? (5) Does the text condemn the perpetrator? (6) Does the text agree to sentence Sun to death? (7) Whether to express dissatisfaction with the government and society? (8) Does the text mention the attempt to quit the doctor's job or express regret about studying medicine? (9) Whether to call for measures to protect doctors? (10) Does the text mention that this incident should be reported more widely? (11) Is the imperfect medical system the reason for this accident? (12) Does the post contain valuable suggestions and measures?

Then, two authors (ND and QQ) used this coding scheme to analyze 542 selected Weibo posts for the final content analysis, and each of them analyzed half of these posts. After the two coders finished their work, they mutually checked 10% of the micro-blogs from each other's subset to ensure consistency in coding. Finally, one author (YX) double-checked a random subset of 10% of all micro-blogs for content analysis to ensure the reliability and accuracy of the coding. Any differences were solved through joint discussions among authors YX, ND, and QQ to reach an agreement. Interrater reliability was evaluated by calculating Cohen kappa, with a value of 0.6 or above indicating adequate reliability. The final Kappa scores ranged from 7.25 to 1.00, indicating substantial to perfect agreement. The researchers conducted a descriptive statistical analysis of the comments on the CAGH incident. We calculated the frequency and proportion of opinions about the CAGH incident according to the user's type (organization or individual) and occupation. We also provided examples of micro-blog posts on each topic. All statistical analyses were performed using SPSS 22.0 and Microsoft Excel 2019.

## Results

### The identity of Weibo users

The majority (89.3%) of posts (*n* = 484) were published by individual users, and the proportion of micro-blogs posted by organizations accounted for 10.7%. With regard to the 58 organizational users, the news media published the most micro-blogs (27.5%), while medical institutions only accounted for 6.8%. The legal service agencies published 15 micro-blogs (25.8%), including three micro-blogs before the judgment date and 12 micro-blogs after the judgment date. Among all individual users, 133 claimed to be medical professionals (27.4%), including 124 doctors, seven nurses and 2 pharmacists. There were only 5 users in the legal profession (1.0%).

### Attitude and opinions about the CAGH incident

Our data shows that 86.7% of posts (*n* = 470) expressed sympathy for the dead doctor. These users used emotional adjectives such as “sad,” “praying” and “crying” to express their feelings. Moreover, 434 micro-blogs (80.0%) condemned the perpetrator, and 26.1% of micro-blogs (*n* = 141) hoped that Sun could be executed or given heavier punishment. However, nearly 20% of micro-blogs did not evaluate the behavior of the perpetrator. We found that two users recalled their experience in CAGH, criticizing the irresponsible attitude of doctors and the poor medical conditions in this hospital. Three other users accused doctors for poor medical performance, and even supported aggressive behavior of patients and their families. A total of 75 posts thought that the CAGH incident had not been widely spread, calling on the government and news media to pay more attention to the violent attacks on medical staff. More than 23.0% of micro-blogs (*n* = 125) believed that the imperfect medical system was the main cause of the accident, and another 20 posts claimed that the poor doctor-patient relationship was the root of the problem. Table 1 shows the opinions of individual and organizational users on the CAGH incident.

**Table 1:** Views of individual Weibo users and organization Weibo users.

**Question**	**Response**	**Organization user (%)**	**Individual user (%)**	**Total (%)**
Whether to show sympathy for the dead doctor?	Yes	43 (74.2)	427 (88.2)	470 (86.7)
	No	15 (25.8)	57 (11.8)	72 (13.3)
Does the text condemn the perpetrator?	Yes	34 (58.6)	400 (82.6)	434 (80.0)
	No	24 (41.4)	84 (17.3)	108 (20.0)
Does the text agree to sentence Sun to death?	Yes	20 (34.5)	121 (25.0)	141 (26.1)
	No	38 (65.5)	363 (70.0)	401 (73.9)
Whether to express dissatisfaction with the government and society?	Yes	2 (3.4)	252 (52.0)	254 (46.8)
	No	56 (96.6)	232 (48.0)	288 (53.2)
Does the text mention the attempt to quit the doctor's job or express regret about studying medicine?	Yes	0 (0.0)	145 (30.0)	145 (26.7)
	No	58 (100.0)	339 (70.0)	397 (73.3)
Whether to call for measures to protect doctors?	Yes	21 (36.2)	156 (32.2)	177 (32.6)
	No	37 (63.8)	328 (67.8)	365 (67.4)
Does the text mention that this incident should be reported more widely?	Yes	6 (10.3)	69 (14.2)	75 (13.8)
	No	52 (89.7)	415 (85.8)	467 (86.2)
Is the imperfect medical system the reason for this accident?	Yes	11 (18.9)	114 (23.5)	125 (23.1)
	No	47 (81.1)	370 (76.5)	417 (76.9)
Does the post contain valuable suggestions and measures?	Yes	19 (32.7)	58 (11.9)	77 (14.2)
	No	39 (67.3)	426 (88.1)	465 (85.8)

All responses are determined according to the content of Weibo.

Among the micro-blogs published before the judgment day, 49.5% of posts (*n* = 248) expressed disappointment with the government and society, while among the micro-blogs published after the judgment day, the proportion was 14.6%. These users argued that the poor protection of doctors was the fault of the Chinese government and the society. Compared with organization users, the proportion of individual users dissatisfied with the government or society is much higher (52.0 vs. 3.4%). Our study shows that 26.7% of micro-blogs (*n* = 145) thought that becoming a health care worker was a bad career choice. Table 2 shows the views of individual users from different backgrounds on the CAGH incident. Among the individual users, 58.6% of HPs (*n* = 78) expressed regret to engage in medical profession, including 75 doctors, two nurses and one pharmacist. Further analysis shows that 59.2% of medical staff users expressed their regret for studying medicine in the posts published before the judgment day, while this proportion dropped to 33.3% in the posts published after the judgment day.

**Table 2:** Views of medical staff Weibo users and other profession Weibo users^#^.

**Question**	**Response**	**Medical staff (%)**	**Other occupations (%)**	**Total (%)**
Whether to show sympathy for the dead doctor?	Yes	112 (84.2)	315 (89.7)	427 (88.2)
	No	21 (15.8)	36 (10.3)	57 (11.8)
Does the text condemn the perpetrator?	Yes	104 (78.1)	296 (84.3)	400 (82.6)
	No	29 (21.9)	55 (15.7)	84 (17.4)
Does the text agree to sentence Sun to death?	Yes	15 (11.2)	106 (30.1)	121 (25.0)
	No	118 (88.8)	245 (69.9)	363 (75.0)
Whether to express dissatisfaction with the government and society?	Yes	90 (67.6)	162 (46.1)	252 (52.0)
	No	43 (32.4)	189 (53.9)	232 (48.0)
Does the text mention the attempt to quit the doctor's job or express regret about studying medicine?	Yes	78 (58.6)	67 (19.0)	145 (29.9)
	No	55 (41.4)	284 (81.0)	339 (70.1)
Whether to call for measures to protect doctors?	Yes	51 (38.3)	105 (29.9)	156 (32.2)
	No	82 (61.7)	246 (70.1)	328 (67.8)
Does the text mention that this incident should be reported more widely?	Yes	23 (17.2)	46 (13.1)	69 (14.2)
	No	110 (82.8)	305 (86.9)	415 (85.8)
Is the imperfect medical system the reason for this accident?	Yes	50 (37.5)	64 (18.2)	114 (23.5)
	No	83 (62.5)	287 (81.8)	370 (76.5)
Does the post contain valuable suggestions and measures?	Yes	21 (15.7)	37 (10.5)	58 (11.9)
	No	112 (84.3)	314 (89.5)	426 (88.1)

Medical staff Weibo users and other profession Weibo users all belong to individual users.

# Other profession Weibo users refer to individual Weibo users except medical staff.

Our data indicates that 32.6% of micro-blogs (*n* = 177) mentioned that effective measures should be taken to prevent hospital violence and strengthen the personal safety protection of health care providers including doctors. Another three posts thought that the safety of nurses is also worthy of attention. There were 24 posts advocating slogans such as “reject violence” or “stop violence.” However, only 14.2% of micro-blogs (*n* = 77) put forward some concrete strategies to prevent hospital violence. We didn't find any constructive measures in the remaining posts. There were seven posts even suggested that doctors should carry knives at work to cope with possible violent attacks (see Table 3).

**Table 3:** Examples of micro-blogs^A^.

**Senders**	**Micro-blog texts**
**Organizations**	
中国之声 Voice of China^1^	#民航总医院内行凶嫌疑人孙文斌被批捕#【#央视热评#:向医生挥刀是彻底的丧心病狂】“急诊科抢救室”,是医生和死神赛跑的地方。对犯罪嫌疑人来说,将暴力加诸救死扶伤的医生,是种彻底的丧心病狂。和悲剧发生的惩治相比,通过有效预防避免犯罪发生更为重要。在医院,决不允许屠刀的存在！ #Sun Wenbin, a suspect who committed a crime in the General Hospital of Civil Aviation, was arrested# [# CCTV hot comment #: Cutting a doctor with a knife is completely insane] The emergency department is a place where doctors race against death. It is crazy and unacceptable for criminal suspects to use violence against doctors who save lives. Compared with punishment after tragedy, it is more important to avoid crime through effective prevention. In hospitals, butchers are never allowed!
重庆检察 Procuratorate of Chongqing^2^	【央视热评:向救死扶伤的医生挥刀是彻底的丧心病狂】一旦犯罪行为导致正常医疗秩序混乱,更多病人将因不能得到及时诊治而成为受害者.从更长远看,当暴力伤医频发让人心有余悸,本有志于从医的优秀人才对医生职业敬而远之的时候,每个人都将是受害者。对暴力伤医行为,严惩是必要的,但仅有严惩是不够的。和悲剧发生的惩治相比,通过更有效预防避免犯罪发生更为重要 [CCTV hot comment: Cutting a doctor with a knife is completely insane] Once the criminal behavior leads to the disorder of normal medical order, more patients will become victims because they can't get timely diagnosis and treatment. In the long run, when violent attacks on doctors occur frequently, which makes talented people unwilling to engage in the medical profession, everyone will become a victim. Severe punishment is necessary for violent attacks on doctors, but severe punishment alone is not enough. Compared with punishment after tragedy, it is more important to avoid crime through effective prevention.
医学界网站 Medical Website^3^	#柳叶刀:保护中国医生#仅靠法律手段难以充分解决医患冲突这一复杂问题。 暴力伤医是一个全球性问题,几乎影响到所有医疗保健机构中的医务工作者。然而,相较之下,中国医务人员遭受暴力伤害的规模、频率和危害性尤为严重。在中国,暴力伤医行为有诸多原因,如基层医疗卫生体系不健全、低效的医患沟通等。因此,通过加强安保措施等方式来修复日益恶化的医患关系收效甚微。若想终止中国的暴力伤医事件,仅通过执法和惩罚性措施是远远不够的,必须要进行就医文化转变,而文化改变需要时间。医疗卫生工作者需要得到信任和尊重,而增强信任最好的方式就是建立一个同样有效、可靠且值得尊重的医疗卫生体系 #Lancet: Protecting Chinese Doctors# It is difficult to completely solve the complicated doctor-patient conflict by legal means alone. Violence against medical staff is a global problem, affecting medical workers in almost all medical institutions. However, the scale, frequency and harmfulness of violence against medical staff in China are particularly serious. In China, there are many reasons for violence against doctors, such as the imperfect primary health care system and inefficient communication between doctors and patients. Therefore, strengthening the safety measures will have little effect on repairing the deteriorating doctor-patient relationship. If we want to stop the violence against doctors in China, it is not enough to just take punitive measures. We must change the medical culture, but it takes time to change it. Medical staff need to be trusted and respected, and the best way to enhance trust is to establish an effective, reliable and respectable health care system
浙江理工大学学生社团联合会 Zhejiang Sci-Tech University Student Association^4^	#民航总医院内行凶嫌疑人孙文斌被批捕#虽然浙理没有医学院,但我们深深知道培养一个医生是多么困难的。24年熬成的主治医生,最后却死在自己患者的刀下。面对高强度的工作,还有日益紧张的医患关系,医生需要社会的关注和帮助！ #Sun Wenbin, a suspect who committed a crime in the General Hospital of Civil Aviation, was arrested #Although there is no medical school in Zhejiang University of Technology, we know how difficult it is to train a doctor. It took Yang 24 years to become a doctor with a high professional title, but she was finally killed by her own patient with a knife. Facing the busy work and increasingly tense doctor-patient relationship, doctors need social attention and help!
Individuals^B^
螺#### snail####^a^	#民航总医院被扎伤女医生去世#我真的大早上太难受了 学医真的救不了中国人 救了很多很多的人,救治者不感激你反而对你拔刀相向,为什么我当初要学医啊,期末考要疯了就算了,以后还会有可能被患者家属杀死,艹[泪][泪][泪][泪][泪]我太难受了,医生不是人吗[泪][泪][泪] #The female doctor who was stabbed in the Civil Aviation General Hospital died# I'm really sad. Doctors can't save Chinese! We saved a lot of patients, but they tried to kill us! Why did I choose to be a doctor in the first place? Not only do I have to go through a long process of studying medicine, but I also have to worry about being killed by patients in the future. I feel terrible. Aren't doctors human beings?
检#### Check####^b^	#民航总医院被扎伤女医生去世#不要再提“一个巴掌拍不响”之类的话了,医生在救死扶伤,这些人在背后捅刀,怎么不让去心寒？为凶手“洗地”就是为他行凶寻找正当性,必须刹住这种风气。伤医,伤的是整个医生群体的心,必须依法严惩！ #The female doctor who was stabbed in the Civil Aviation General Hospital died # Don't mention the words “two hands are needed to make a sound”. Doctors save lives, but patients and their families want to kill doctors with knives. How can such behavior not make people feel chilling? It is unacceptable to defend the murderer. This trend must be stopped. Attacking doctors violently will make all medical staff feel pain and helplessness. The killer must be severely punished according to law!
Miyu#### Miyu####^c^	#民航总医院被扎伤女医生去世#建议医生学点防身术,办公室里藏把刀,关键时刻保证自己是见法官而不是见判官的。 #The female doctor who was stabbed in the Civil Aviation General Hospital died # I suggest that all doctors learn self-defense and hide a knife in their office to protect themselves.This can ensure that you will see the judge rather than God at a critical moment
Lancy#### Lancy####^c^	#民航总医院被扎伤女医生去世#什么叫被扎伤？！？都用刀割了！！真的很心痛！怎么会已经杀了人,家属还若无其事地继续给医生施压要求治疗的呢？不仅是凶手还有那些冷漠的、只关心自己治疗的人！人性还是依旧这么丑陋吗？！我曾经一度想学医高考也是这个目标,我妈是医生她坚决不同意,现在我可能是知道原因了。看着身边学医的同学一到期末就有十几本厚书要背,我还嘲笑她我们都毕业工作了她还要读书。想想以后,又有多少人愿意学医呢？这个世界还会好吗？ #The female doctor who was stabbed in the Civil Aviation General Hospital died# Why does the news use the word stab wound?! Apparently, the killer cut the doctor's head with a knife!! I feel very sad! One of the patients' family members killed Dr. Yang, but other family members asked the doctors to continue to treat the patient, instead of caring for Dr. Yang's safety. Why don't other patients and their families help Dr. Yang? Human nature is still so ugly!? I wanted to study medicine in high school, but my mother strongly disagreed. Now I know the reason. Medical students have a lot of knowledge to memorize every term. The training period of medical students far exceeds that of other majors. However, how many people are willing to be doctors in the future? Will our society be better?
Ci#### Ci####^c^	#民航总医院被扎伤女医生去世#我心里好痛啊。不知道救治过多少病患的医生,就这么被渣渣轻飘飘一刀捅没了？到底还要牺牲多少位在临床一线辛苦奋战的医护人员才能引起政府和社会的重视啊？ #The female doctor who was stabbed in the Civil Aviation General Hospital died # My heart hurts so much. The doctor saved so many lives, but he was killed by an inhuman patient's family. How many hard-working medical staff do we have to sacrifice in order to attract the attention of the government and society?

A Translated by the authors.

B Authors anonymized the user's Weibo names for privacy concerns.

1 News media.

2 Legal institution.

3 Medical institution.

4 Other organization.

a Doctor.

b Lawyer.

c Other occupations.

## Discussion

No matter before or after the outbreak of COVID-19, personal attacks and daily insults in the workplace are increasing (4), but hospital violence is still largely ignored and underestimated (15). Our results show that both news media organizations and legal service organizations actively disseminate information about the CAGH incident through Weibo. However, organizational users were not the only source of information on medical WPV. Individual Weibo users such as doctors and nurses also played a role in spreading information. Our analysis also found that in addition to promoting the dissemination of information, individual users also actively participated in the topic discussion about preventing hospital violence and protecting doctors. This finding is consistent with existing research on social networks, which found that social media has become an important medium for information dissemination and is playing a unique role in information sharing and health care discussion (16).

In this study, the victim was a doctor from the emergency department of a tertiary hospital. Previous studies have identified environment, staff and patient risk factors as the main precursors of WPV initiated by patients (17). Ma et al. (18) found that serious WPV mainly occurred in cities (90.2%), usually in tertiary hospitals (67.9%) (18). One possible explanation is that tertiary hospitals in China usually have better equipment and doctors, and patients with serious diseases usually seek help (19). This means that the annual death toll in tertiary hospitals may be higher, thus increasing the risk of serious WPV. A recent systematic review pointed out that aggression is more common in departments where workers come into contact with patients with mental illness or drugs, such as emergency rooms (4). However, there is no workplace or occupational category that does not face this risk (4). Generally speaking, nurses are more vulnerable to verbal violence than doctors (20), but doctors are more often victims of physical violence in the workplace (21). As doctors are the designers and practitioners of medical diagnosis and treatment programs, if patients and their families are dissatisfied with the medical procedures, results or quality, doctors are most likely to be the targets of WPV. Besides, as our research showed, the perpetrators are usually the patients' families, not the patients themselves (22, 23). This phenomenon has been reported in different periods (including COVID-19 period) and different cultures (23–25). This may be because patients sometimes can't move or fight due to age and medical conditions. Relatives may express their dissatisfaction, anger or economic intentions (compensation) through violence.

Compared with previous studies (26), the legal service institutions in this research were more active in discussing medical WPV. However, the legal opinions on the CAGH incident were mainly concentrated after the trial. In other words, users from the legal profession did not intervene in the discussion in time after the hospital violence occurred, which may be related to the informal nature of social media. We must realize that under the background of hospital violence, many legal issues related to workplace safety need to be seriously solved (6). The 2007 European Agreement on harassment and violence at work has indicated WPV as a particular psychosocial risk factor (27), which should be tackled by employers through evidence-based interventions, established in the framework of the mandatory risk assessment process required by occupational health and safety (OHS) laws (1). Before the CAGH incident, there were no special legal documents in China to strengthen the protection of HPs in medical workplaces. On December 28th, 2019, the third day after the CAGH incident, China's highest legislature passed the Law of the People's Republic of China on the Promotion of Basic Medical and Health Care, which was the first law in China to protect HPs and had epoch-making significance. In addition, in the International Labor Organization 2020 report, all the countries have been invited to incorporate provisions related to workplace violence into their occupational safety and health laws, and formulate specific guidelines and standards to support the implementation of plans and preventive measures in the workplace (28).

Our research showed that 20% of posts did not even condemn the perpetrator, which indicates that some users may have a tolerant attitude toward hospital violence (26). Many users condemned the government's lack of effective protection measures and asked the society to give HPs more respect. A Weibo user complained, “How many hard-working medical staff do we have to sacrifice to attract the attention of the government and society?” The emotional impact of WPV on HPs is grave, with many HPs exposed to high levels of mental stress and increased predisposition to mental illness alongside thoughts of quitting their engagement as HPs (4). We found that nearly 60% of HPs users expressed their fear and regret about engaging in the medical career. Compared with European countries, the number of health care workers per 1,000 population in China is low, which leads to great pressure and workload for front-line clinicians (19). In addition, patients and their families are often dissatisfied with the crowded medical environment, long waiting time, and insufficient communication with clinicians (19, 29). Such a trend reduces the quality of HPs' work, and may increase their burnout and turnover intention (4). Because of the fear of an unsafe working environment, the popularity of studying medicine among young people is decreasing, which may increase the turnover rate of registered clinicians (30). The shortage of HPs will increase the workload of existing hospital staff, thus reducing the quality of care and perpetuating the cycle of violence against HPs (19). A valuable discovery is that after the criminal was sentenced to death, HPs' willingness to leave their jobs decreased, which indicates that severe sanctions from the legal level may help improve the professional confidence of HPs.

In this study, one-third of users called for effective measures to protect HPs and stop hospital violence, but most posts did not contain any valuable suggestions. We found that seven posts even suggested that doctors should carry knives with them to protect themselves in the face of long-term WPV. This proposal is a little extreme, but it may expose the challenge that the current measures to prevent hospital violence have little effect. Besides its impact on HPs, WPV will also damage the accessibility and quality of the health system, and may lead to public dissatisfaction with the government (31). Previous literature showed that the Chinese government has tried to reduce the risk of deterioration of doctor-patient relationships through medical and educational reforms, such as training family doctors to develop a strong primary health system (32), and establishing a standardized resident training system to improve the ability of young doctors (33). Although some achievements have been achieved, there are still many challenges to solve the problem of WPV against HPs. Experience has told us that there is no single strategy to solve this problem due to the complexity of hospital violence. Only by coordinating the implementation of structural, organizational and individual interventions, preferably participatory interventions, can effective results be achieved (34). First of all, the management of hospitals should develop communication strategies through which information on delays in service provision during long waiting times are properly communicated to patients and their relatives (4). Second, provide enough staff to reduce the weekly working hours of individual medical staff, or make education and training plans to help HPs manage WPV better (9). Third, interpersonal support should be promoted in professional groups. Finally, it may also be beneficial to raise public awareness of the negative impact of medical WPV through mass media publicity, and to implement appropriate legislation and policies (eg, judicial punishment for the perpetrators).

A long-term impact of the hospital violence on the healthcare industry would be far-reaching. Faced with China's growing and aging population, as well as the shortage of doctors and the low quality of medical care, the health care crisis is foreseeable. More challenging, this health crisis may not be limited to China. According to the latest survey (4), the COVID-19 epidemic may increase the medical WPV around the world, especially because of a heavy workload, stressful work settings and insufficient medical resources. In a word, medical WPV and HPs' occupational safety deserve continuous dialogue and careful examination.

### Limitations

There are a few limitations in this study. First, although social media data may reflect a wider public perspective, it also has its bias and limitations. It should not be regarded as the full representation of societal opinion, but a supplement to other information media. Future research can focus on other social platforms to compare with our study. Second, the micro-blogs we searched were all original micro-blogs, that is, we didn't record the micro-blogs forwarded by users, which made us unable to obtain all micro-blogs related to the CAGH incident. However, the micro-blogs forwarded by users usually can't fully express the users' own views, so we think the impact is limited. Third, the content of some micro-blogs is very short, so it can't completely correspond to the codes we set, which has some adverse effects on the accuracy of our research results. Finally, WPV against HPs may have long-term effects, which requires more future studies.

## Conclusion

Social media is an important tool for gauging reaction to health events. Both organizational and individual users on Weibo actively participated in the dissemination and discussion of the CAGH incident, but legal opinions failed to intervene in time. Most posts expressed sympathy for the dead doctor, but the effective measures to prevent medical WPV were rarely mentioned. Serious hospital violence may increase public dissatisfaction with the government and the tendency of medical staff to leave. The complexity of hospital violence determines that only the coordinated implementation of structure, organization and individual intervention can achieve effective results.

## Data availability statement

The raw data supporting the conclusions of this article will be made available by the authors, without undue reservation.

## Author contributions

Data curation: YX, ND, and Q-mQ. Investigation: JC. Writing—original draft: YX. Writing—review and editing: Y-lL and S-yZ. All authors contributed to the article and approved the submitted version.

## Conflict of interest

The authors declare that the research was conducted in the absence of any commercial or financial relationships that could be construed as a potential conflict of interest.

## Publisher's note

All claims expressed in this article are solely those of the authors and do not necessarily represent those of their affiliated organizations, or those of the publisher, the editors and the reviewers. Any product that may be evaluated in this article, or claim that may be made by its manufacturer, is not guaranteed or endorsed by the publisher.

## Abbreviations

CAGH: Civil Aviation General Hospital
WPV: workplace violence
HPs: health professionals.
